# Emergence of a mutation in the nucleocapsid gene of SARS-CoV-2 interferes with PCR detection in Canada

**DOI:** 10.1038/s41598-022-13995-4

**Published:** 2022-06-27

**Authors:** Sandra Isabel, Mariana Abdulnoor, Karel Boissinot, Marc R. Isabel, Richard de Borja, Philip C. Zuzarte, Calvin P. Sjaarda, Kevin R. Barker, Prameet M. Sheth, Larissa M. Matukas, Jonathan B. Gubbay, Allison J. McGeer, Samira Mubareka, Jared T. Simpson, Ramzi Fattouh

**Affiliations:** 1 Department of Laboratory Medicine and Pathobiology, Temerty Faculty of Medicine, University of Toronto, Toronto, ON Canada; 2 Public Health Ontario, Toronto, ON Canada; 3 Division of Microbiology, Department of Laboratory Medicine, St. Michael’s Hospital, Unity Health Toronto, 30 Bond Street, Toronto, ON M5B 1W8 Canada; 4 Ontario Institute for Cancer Research, Toronto, ON Canada; 5 Queen’s Genomics Lab at Ongwanada (Q-GLO), Kingston, ON Canada; 6 Department of Psychiatry, Queen’s University, Kingston, ON Canada; 7 Division of Microbiology, Department of Laboratory Medicine and Genetics, Trillium Health Partners, Mississauga, ON Canada; 8 Department of Pathology and Molecular Medicine, Queen’s University, Kingston, ON Canada; 9 Division of Microbiology and Infectious Diseases, Kingston Health Sciences Center, Kingston, ON Canada; 10 Department of Biomedical and Molecular Sciences, Queen’s University, Kingston, ON Canada; 11 Hospital for Sick Children, Toronto, ON Canada; 12 Lunenfeld-Tanenbaum Research Institute, Sinai Health System, Toronto, ON Canada; 13 Sunnybrook Research Institute, Toronto, ON Canada; 14 Department of Computer Science, University of Toronto, Toronto, ON Canada; 15 Li Ka Shing Knowledge Institute, Unity Health Toronto, Toronto, ON Canada; 16 Institute for Better Health, Trillium Health Partners, ON Mississauga, Canada; 17 Matane, Quebec, Canada

**Keywords:** Phylogenetics, Infectious diseases

## Abstract

The emergence of Severe Acute Respiratory Syndrome Coronavirus (SARS-CoV-2) was met with rapid development of robust molecular-based detection assays. Many SARS-CoV-2 molecular tests target multiple genetic regions of the virus to maximize detection and protect against diagnostic escape. Despite the relatively moderate mutational rate of SARS-CoV-2, numerous mutations with known negative impact on diagnostic assays have been identified. In early 2021, we identified four samples positive for SARS-CoV-2 with a nucleocapsid (N) gene drop out on Cepheid Xpert® Xpress SARS-CoV-2 assay. Sequencing revealed a single common mutation in the N gene C29200T. Spatiotemporal analysis showed that the mutation was found in at least six different Canadian provinces from May 2020 until May 2021. Phylogenetic analysis showed that this mutation arose multiple times in Canadian samples and is present in six different variants of interest and of concern. The Cepheid testing platform is commonly used in Canada including in remote regions. As such, the existence of N gene mutation dropouts required further investigation. While commercial SARS-CoV-2 molecular detection assays have contributed immensely to the response effort, many vendors are reluctant to make primer/probe sequences publicly available. Proprietary primer/probe sequences create diagnostic ‘blind spots’ for global SARS-CoV-2 sequence monitoring and limits the ability to detect and track the presence and prevalence of diagnostic escape mutations. We hope that our industry partners will seriously consider making primer/probe sequences available, so that diagnostic escape mutants can be identified promptly and responded to appropriately to maintain diagnostic accuracy.

## Introduction

Severe Acute Respiratory Syndrome Coronavirus 2 (SARS-CoV-2) was identified as the etiological agent of coronavirus disease (COVID-19) with initial cases documented in Wuhan, China, in December 2019. A pandemic was declared in March 2020, and more than 200 million cases have been documented worldwide as of October 2021^1^. SARS-CoV-2 is an RNA virus with a mutation acquisition rate estimated at 1.1 × 10^–3^ subs/site/year^2^. PCR-based diagnostic assays have been at the forefront of identification of COVID-19 cases and have played an integral role in the public health efforts to control transmission. Academic and industry groups alike have designed and produced many rRT-PCR assays to detect different genes of SARS-CoV-2.

In the winter of 2021, two nasopharyngeal swab (NPS) specimens tested with the Cepheid Xpert® Xpress SARS-CoV-2 assay in our institution produced an unusual pattern of detection, with low cycle threshold (Ct) values (between 13 and 15) for the envelope (E) gene target and nucleocapsid (N) gene ‘not detected’. We performed whole genome sequencing (WGS) to study the reasons leading to the N gene drop out and identified only one common mutation (synonymous C29200T) in the N gene of those two specimens (EPI_ISL_1490673 and EPI_ISL_1490676). Analysis of whole genome sequences previously generated from SARS-CoV-2-positives identified by our laboratory found two additional specimens harbouring the C29200T mutation (EPI_ISL_1490692 and EPI_ISL_1490679). These two specimens had been originally tested with another rRT-PCR assay (AltoStar SARS-CoV-2 assay; targets the spike (S) and E genes) and resulted in similarly low Ct values for the E gene (Ct 15.1 and 17.1) with N gene not detected when a 1:10 dilution was tested on the Xpert® Xpress SARS-CoV-2 PCR assay. These four samples were diluted 1:10 due to the limited specimen volumes and subsequently tested on the BD SARS-CoV-2 Reagents for BD MAX™ System; both N1 and N2 targets were detected with Ct values ranging from 11.4–16.7 and 12.4–17.0, respectively. Two of these four SARS-CoV-2-positive samples (EPI_ISL_1490673 and EPI_ISL_1490679) were also tested on the Xpert® Xpress SARS-CoV-2/Flu/RSV Assay, which combines E and N2 targets in a single channel and, expectedly, tested positive with Ct values of 12 and 15. However, we could not determine if the C29200T mutation caused an N gene drop out in the Xpert® Xpress SARS-CoV-2/Flu/RSV assay.

**Figure 1:**
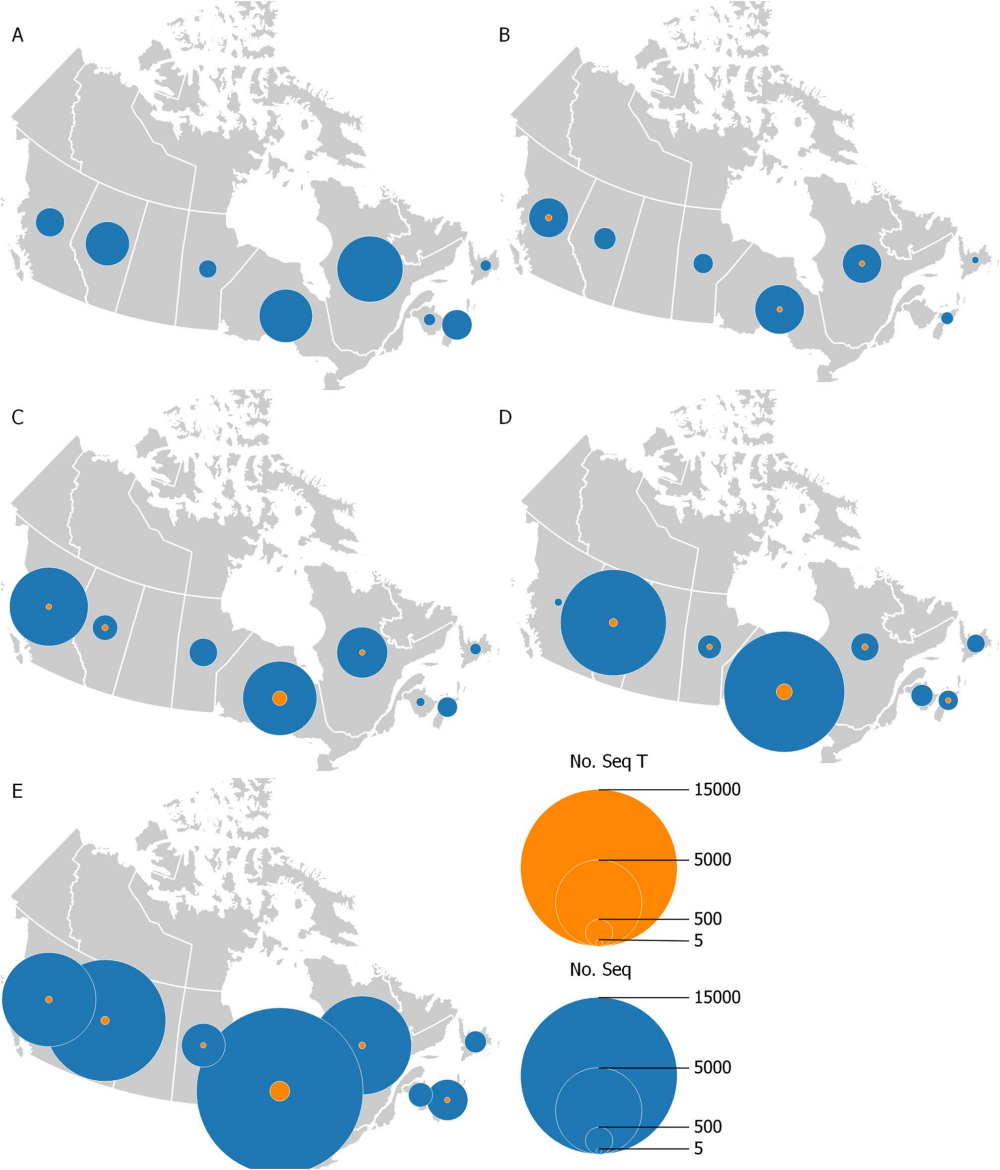
Canadian distribution of SARS-CoV-2 genome sequences with the C29200T mutation. Blue circles represent the number of SARS-CoV-2 sequences per province or territory per time period available on GISAID. The orange circles indicate the number of sequences including the C29200T mutation. Data were retrieved from GISAID as of May 22, 2021, and of samples collected from (**A**) January–April 2020, (**B**) May–August 2020, (**C**) September–December 2020, (**D**) January–April 2021, and (**E**) January 2020-April 2021. The maps were built with the geographic information system QGIS (v2.18.21, https://qgis.org).

We then evaluated the distribution of the C29200T mutation in Canada. We analysed all 41,712 SARS-CoV-2 genomes from Canada available on GISAID as of May 22nd, 2021, and found 266 sequences with the C29200T mutation (0.6%), 7 sequences with Y ambiguities (meaning C or T), and 794 sequences with indeterminate base pair (N) at that position (Fig. 1). SARS-CoV-2 sequences from eight provinces were downloaded from GISAID with six provinces having at least one sequence with the C29200T mutation. Of those provinces, Ontario had the highest number of genomes with the C29200T mutation in 241 of 16,772 sequences (1.4%). Alberta, British Columbia, Nova Scotia, Manitoba, and Quebec had a lower number of sequences with the C29200T mutation ranging from 1 to 15 (0.1–0.2%) (Table 1). By the end of April 2021, approximately 1.2 million infections had been documented in Canada and we analysed sequences for 3.5% of those cases^3^. Therefore, the frequency presented above could be inaccurate. We then analysed the spatiotemporal distribution of the SARS-CoV-2 C29200T mutation and found the first sequences in Canada in the spring of 2020, and an increase in the number and proportion of sequences with this mutation in the fall and winter of 2020/2021, particularly in Ontario (Fig. 1). Because specimens sequenced and uploaded to GISAID are not known to be representative, we did not further analyze the mutation frequency by geography or time period (Table 1).

**Table 1:** Number of SARS-CoV-2 sequences with C29200T mutation and total number of sequences in Canada.

Province	Jan–Apr 2020		May–Aug 2020		Sep–Dec 2020		Jan–Apr 2021		Jan 2020–April 2021		
	C29200T	No. Seq	C29200T	No. Seq	C29200T	No. Seq	C29200T	No. Seq	Total C29200T	Total No. Seq	Total % of C29200T
Alberta	0	1367	0	311	2	425	13	7328	15	9431	0.2
British Columbia	0	568	3	1095	1	4202	0	11	4	5876	0.1
Manitoba	0	189	0	245	0	541	1	369	1	1344	0.1
New Brunswick	0	60	0	0	0	19	0	303	0	382	0.0
Quebec	0	3007	1	1071	1	1817	2	530	4	6425	0.1
Ontario	0	1989	1	1709	104	3752	136	9322	241	16,772	1.4
New Foundland/Labrador	0	47	0	6	0	49	0	199	0	301	0.0
Nova Scotia	0	622	0	69	0	248	1	242	1	1181	0.1
Total	0	7849	5	4506	108	11,053	153	18,304	266	41,712	0.6

**Figure 2:**
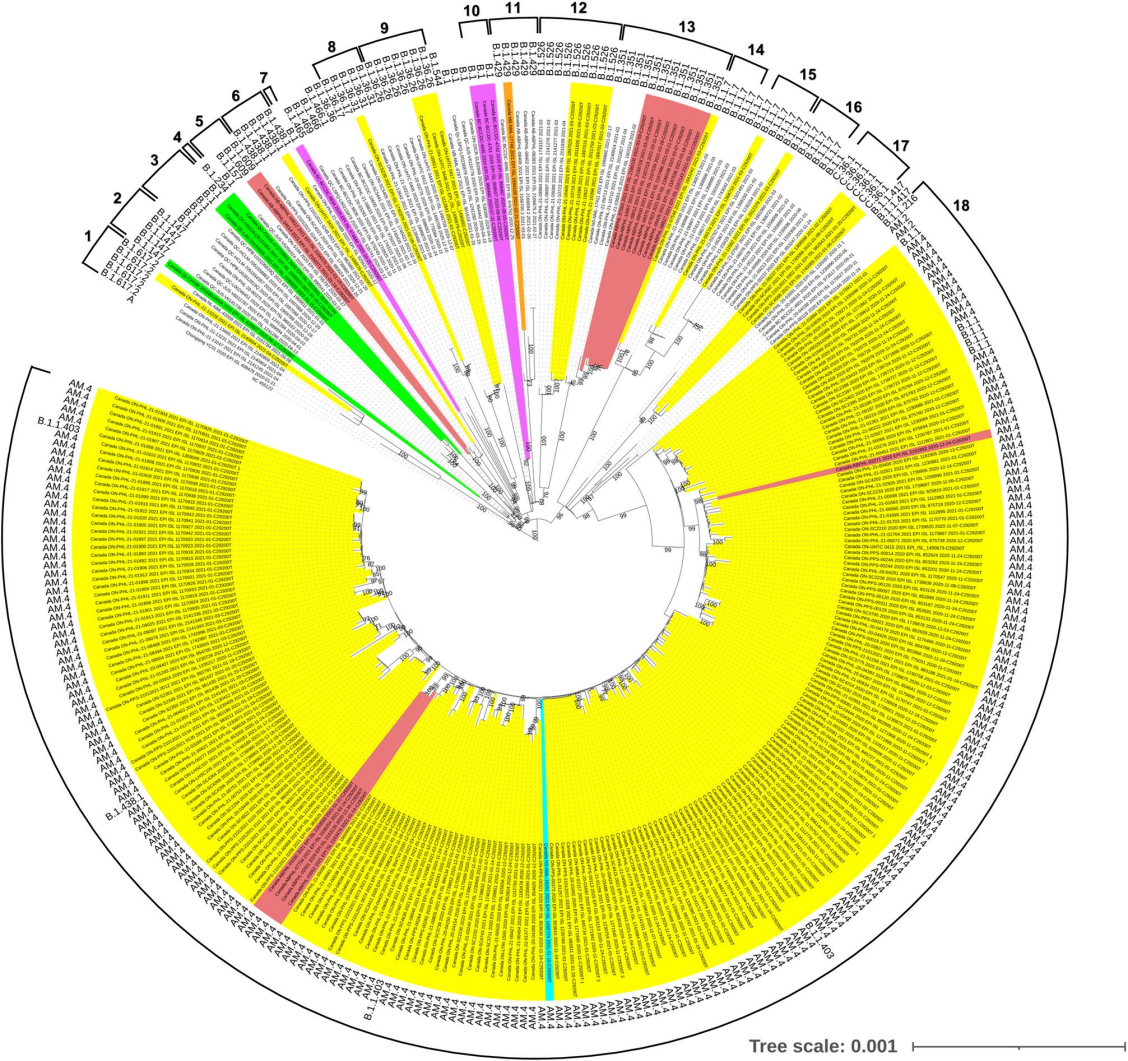
Maximum-likelihood tree of 337 sequences of SARS-CoV-2 in Canada and emergence of C29200T clades. Colour codes show the province where the patient sample was collected for the strains with C29200T mutations as follows: Alberta in red, British Columbia in magenta, Manitoba in orange, Nova Scotia in light blue, Ontario in yellow, and Quebec in lime. Sequences without the mutation used as controls were left uncolored. Sequence lineages are also indicated. The sixteen clades (#1–3, 5–6, 8–18) and two singletons (#4 and 7) analysed are identified with the outer numbers.

Finally, we performed phylogenetic analyses to study genetic relatedness in Canada and identified 16 different clades and two singletons with the C29200T mutant strains including a clade with 230 sequences (Fig. 2). The main clade (#18) included 224 sequences from Ontario, five from Alberta and one strain from Nova Scotia from November 2020 to January 2021. The sequence from Nova Scotia (EPI_ISL_1055724) and five sequences from Alberta are closely related to the strains from Ontario. There are two standalone Alberta clades with two and eight sequences (#5 and #13). There were nine other smaller clades in Ontario (clades #1, 6, 8–9, 12, 14–17) each including one to five sequences with C29200T mutation with other closely related sequences without that mutation. Of the four sequences from British Columbia, three cluster together (July 2020) and one is different (October 2020). The sequences from Quebec are located on three different branches and cluster mainly with sequences from Quebec without the C29200T mutation. The single Manitoba sequence is included in a clade of closely related sequences without the mutation C29200T from British Columbia and Alberta. Singletons #4 and #7 include one taxon from Quebec and British Columbia, respectively. Furthermore, we found 19 sequences with the C29200T mutation in six different lineages that have been identified as of variants of interest (VOI; B.1.429 and B.1.526) or variants of concern (VOC; B.1.1.7, B.1.351 and B.1.617.2) (Table 2)^4^. Additionally, we downloaded sequences from GISAID with submission dates between (i) May 25, 2021–September 3, 2021, that are of the ‘B.1.617.2 + AY.*’ (Delta) lineage, and (ii) up to Apr 13, 2022, that are of the ‘B.1.1.529 + BA.*’ (Omicron) lineage, as those lineages predominated in Canada during those times, to examine the presence of the N gene C29200T mutation. In total, 11,092 Delta and 68,535 Omicron sequences were reviewed and the C29200T mutation was found in 4 and 23 sequences, respectively. These analyses showed that this synonymous mutation can, and continues to, arise as independent events and then can be spread through transmission, even interprovincially.

**Table 2:** SARS-CoV-2 lineages harboring C29200T mutation in Canada from data retrieved on May 22, 2021.

Lineage	No. of C29200T
AM.4	222
B.1.351 (Beta)	8
B.1	6
B.1.526 (Iota)	5
B.1.1	4
B.1.1.403	3
B.1.147	1
B.1.1.7 (Alpha)	3
B.1.234	1
B.1.36.26	3
B.1.36.31	1
B.1.429 (Epsilon)	1
B.1.438.1	1
B.1.609	2
B.1.617.2 (Delta)	1
C.36.1	4
Total	266

Previous studies also identified some samples with an N gene escape on the Cepheid Xpert® Xpress SARS-CoV-2 assay^5–8^. Sequencing and RT-PCR on amplicons allowed Ziegler et al*.* to identify the mutation C29200T resulting in N gene detection escape. That mutation anneals in the centre of the CDC-N2 probe leading them to hypothesize that the Xpert® Xpress SARS-CoV-2 assay used that design^6^. Miller et al. sequenced the Xpert SARS-CoV-2 amplification products (positions 29164 to 29230) and identified two N gene mutations (C29197T and C29200T) that caused a Ct shift and/or loss of N gene amplification^8^. Leelawong et al. found five strains displaying an N gene drop out on the Xpert® Xpress SARS-CoV-2 assay with the common mutation C29200T^5^. However, when they used another CDC N2 gene-based assay they found that both the E and N gene-targets were well amplified. This led Leelawong et al. to question whether a molecular beacon (instead of a TaqMan probe) could result in the N gene drop out in the Xpert® Xpress SARS-CoV-2 assay. That our analysis also found strong amplification of the N1 and N2 genes using the BD SARS-CoV-2 Reagents for BD MAX™ System further supports the hypothesis proposed by Leelawong et al. Despite these investigations, a definitive understanding of the question at hand will ultimately require knowledge of the primer and probe sequences utilised in the Xpert® Xpress SARS-CoV-2 assay. In our experience, circumstance (i.e. identification of 2 disparate strains with a single common N gene mutation with 2 additional samples discovered subsequently by sequencing) aided in the identification of the C29200T mutation as the cause of the N gene drop out. While critical in these findings, WGS would have been insufficient on its own to definitively identify a causative mutation had circumstances been different and viral target drop-out had been observed in a single SARS-CoV-2 positive sample. Notably, the Food and Drug Administration has added the Xpert® Xpress SARS-CoV-2 assay to their list of molecular tests impacted by SARS-CoV-2 mutations^9^. C29200T is a synonymous mutation that arose multiple times in evolution and was previously found in 0.2% of the sequences on GISAID from at least five countries^5^. Our study supports and extends the findings of others as we show that this mutation arose multiple times in SARS-CoV-2 evolution, can be spread by transmission, and is found in different lineages including those designated as VOIs and VOCs. Moreover, this is the first report detailing the distribution and emergence of this mutation within Canada.

The existence of recurring mutations that affect PCR efficiency highlights the need for increased transparency around commercial primer/probe sequences. Viral target redundancy has been a bedrock principle of molecular-based diagnostic assays for SARS-CoV-2 in an effort to minimize the potential for false negative results. The C29200T mutation found worldwide results in escape of N gene detection by the Cepheid Xpert® Xpress SARS-CoV-2 assay. This assay has been employed in many settings including tertiary and community hospitals, as well as remote community non-health care settings, owing to the minimal hands-on time requirement, quick turn around and high performance. Co-emergence of another mutation impacting the amplification of the E-gene could fully jeopardize the assay. Importantly however, loss of diagnostic performance is not limited to instances of complete diagnostic escape. Often, analytical sensitivity is not equivalent amongst the viral genes being targeted by a given assay and can differ by orders of magnitude^10^. As a result, loss of even a single gene target can significantly impact assay performance and increase the potential for false negatives. As such, the identification of SARS-CoV-2 strains carrying N gene escape mutations represents a major concern for a diagnostic platform that is highly relied upon in some of the most vulnerable of settings. Even more concerning is the emergence of such mutations amongst SARS-CoV-2 strains designated as VOI’s and VOC’s. Emergence of partial diagnostic escape mutants is not unique to the Xpert® Xpress SARS-CoV-2 PCR assay as other commercial assays have also been affected^11–15^. Inevitably, diagnostic escape mutations will continue to emerge as SARS-CoV-2 evolves. Of immense importance is the ability to identify and track the emergence of such mutations to mitigate diagnostic vulnerabilities in real time and not in retrospect. While commercial assays have been an invaluable pillar in response to the pandemic, many, but not all, vendors have obstructed line of sight towards diagnostic escape under the veil of proprietary knowledge. For this type of information to be withheld amid a pandemic that has claimed the lives of millions and infected hundreds of millions more is a major disappointment, at minimum. In this day and age, the rationale for withholding primer/probe sequences seems limited. In an era where molecular cloning and sequencing are routine methodologies, reverse engineering primer and probe sequences is possible for those who wish to do so. Moreover, primer/probe sequences are not the sole determinant of assay performance. Furthermore, for many clinical laboratories the decision to employ a (particular) commercial assay is complex, driven by numerous considerations and rarely influenced by a lack of knowledge of high-performing primer/probe sequences. Major efforts around the globe have been made to routinely sequence the SARS-CoV-2 genome, and for these sequences to be shared world-wide. In addition, various open-source initiatives have been launched to automate the identification of SARS-CoV-2 mutations that could impact test performance. However, primer/probe sequences that are proprietary create a diagnostic ‘blind spot’ for such initiatives. We call upon our industry partners, and our regulatory authorities, to work towards uncovering these blind spots.

## Materials and methods

### Sequence data, demographics, maps and statistics

A total of 41,712 genomes of SARS-CoV-2 (excluding non-human sequences) and demographics when available were obtained from GISAID (downloaded on May 22, 2021 http://gisaid.org/) to perform the statistical analyses of the C29200T mutation. The dataset included sequences representing eight Canadian provinces. The geographical maps of Canada were built with the geographic information system QGIS (v2.18.21, https://qgis.org).

### PCR and sequencing

Cepheid Xpert® Xpress SARS-CoV-2 Assay (E gene and N gene; Cepheid, Sunnyvale, CA) and BD SARS-CoV-2 Reagents for BD MAX™ System (N(1) and N(2) gene; BD, Franklin Lakes, NJ) testing was performed on nasopharyngeal swabs according to the manufacturer’s instructions using samples EPI_ISL_1490673, EPI_ISL_1490676, EPI_ISL_1490692, and EPI_ISL_1490679. Due to limited sample volumes after clinical PCR testing, samples EPI_ISL_1490692 and EPI_ISL_1490679 were diluted 1:10 in COBAS PCR media prior to Xpert® Xpress SARS-CoV-2 PCR testing. All samples were diluted 1:10 in COBAS PCR media prior to BD Max rRT-PCR testing. No samples were diluted prior to extraction for sequencing. NEBNext ARTIC SARS-CoV-2 Companion Kit (Oxford Nanopore Technologies®) (New England Biolabs, Ipswich, MA) in conjunction with the Native Barcoding Expansion 96 kit, Sequencing Auxilliary Vials (EXP-AUX001), Flow Cell Priming Kit and MinION instrument with R9.4.1 flow cell (Oxford Nanopore Technologies®, Oxford, United Kingdom) were used for sample/library preparation and sequencing as previously described^16,17^.

Bioinformatic analyses were performed using the Artic Network Pipeline v1.1.3 using Nanopolish for variant calling^18^. QC was subsequently performed using Ncov-tools v1.5 with default config.yaml settings except for genome completeness which was set at 0.90^19^.

### Molecular evolution

A total of 41,712 SARS-CoV-2 genomes were obtained from GISAID (downloaded on May 22, 2021). Multiple alignment was performed with MAFFT (v.7.309) and sequences with C29200T mutation were identified. A maximum likelihood tree with IQ tree (v.1.6.2, http://www.iqtree.org/) using HKY substitution model and 1,000 ultrafast bootstraps was created and edited on Itol (v.6.3, https://itol.embl.de/). All Canadian sequences with the C29200T mutation were included as well as 70 closely related sequences without the C29200T mutation as controls. Positions analysed were 345 to 29,661 based on reference sequence used NC_045512 (GenBank). All sequences used in our analyses can be found in GISAID and accession numbers can be found in Fig. 2 and Supplemental Table 1. Additionally, we downloaded all sequences uploaded to GISAID with submission dates of (i) May 25–September 3, 2021, of the Delta (GISAID variant field = ‘B.1.617.2 + AY.*’) lineage and (ii) all up to April 13, 2022, that are of the Omicron (GISAID variant field = ‘B.1.1.529 + BA.*’) lineage. We created an alignment using MAFFT and searched for the C29200T mutation.

### Ethics statement

Specimens were collected from patients by healthcare workers of Unity Health Toronto for testing as part of routine clinical service and informed consent was obtained from all participants and/or their legal guardians. Whole genome sequencing of SARS-CoV-2 positive samples was approved by the St-Michael’s Hospital Research Ethics Board (REB# 94-103) and did not require additional patient’s consent. All methods were carried out in accordance with relevant guidelines and regulations. To protect patient privacy and confidentiality, data are aggregated and reported in an anonymized format.

## Supplementary Information

Supplementary Table 1.

## References

[1] Center for Systems Science and Engineering at Johns Hopkins University. COVID-19 Dashboard. https://coronavirus.jhu.edu/map.html. Accessed 14 Aug 2021 (2021).

[2] Duchene S (2020). Temporal signal and the phylodynamic threshold of SARS-CoV-2. Virus Evol..

[3] Canada COVID-19 situation update. https://www.canada.ca/en/public-health/services/diseases/2019-novel-coronavirus-infection.html?utm_campaign=hc-sc-phm-21-22&utm_medium=sem&utm_source=ggl&utm_content=ad-text-en&utm_term=covid19cases&adv=2122-0008&id_campaign=396443591&id_source=1251244778444200&id_content=&gclsrc=aw.ds&&gclid=076d528fb1d21d9e97a0338fd35d09c8&gclsrc=3p.ds. Accessed 12 May 2021 (2021).

[4] World Health Organization. Tracking SARS-CoV-2 variants. https://www.who.int/activities/tracking-SARS-CoV-2-variants. Accessed 9 May 2022 (2022). 37184162

[5] Leelawong M (2021). SARS-CoV-2 N gene mutations impact detection by clinical molecular diagnostics: Reports in two cities in the United States. Diagn. Microbiol. Infect. Dis..

[6] Ziegler K (2020). SARS-CoV-2 samples may escape detection because of a single point mutation in the N gene. Eurosurveillance.

[7] Rhoads DD (2021). Endemic sars-cov-2 polymorphisms can cause a higher diagnostic target failure rate than estimated by aggregate global sequencing data. J. Clin. Microbiol..

[8] Miller S, Lee T, Merritt A (2021). Single-point mutations in the N gene of SARS-CoV-2 adversely assay. Microbiol. Spectrum.

[9] *SARS-CoV-2 Viral Mutations: Impact on COVID-19 Tests*. https://www.fda.gov/medical-devices/coronavirus-covid-19-and-medical-devices/sars-cov-2-viral-mutations-impact-covid-19-tests. Accessed 3 June 2021 (2021).

[10] LeBlanc JJ (2020). Real-time PCR-based SARS-CoV-2 detection in Canadian laboratories. J. Clin. Virol..

[11] Brown KA (2021). S-Gene target failure as a marker of variant B.1.1.7 among SARS-CoV-2 isolates in the Greater Toronto area, December 2020 to March 2021. JAMA J. Am. Med. Assoc..

[12] Artesi M (2020). CoArt 02.Artesi a recurrent mutation at position 26340 of SARS-CoV-2 is associated with failure of the e gene quantitative reverse. J. Clin. Microbiol..

[13] Wang R, Hozumi Y, Yin C (2020). Mutations on COVID-19 diagnostic targets. Genomics.

[14] Sánchez-Calvo JM, AladosArboledas JC, Ros Vidal L, de Francisco JL, López Prieto MD (2021). Diagnostic pre-screening method based on N-gene dropout or delay to increase feasibility of SARS-CoV-2 VOC B.1.1.7 detection. Diagn. Microbiol. Infect. Dis..

[15] Vanaerschot M (2021). Identification of a polymorphism in the N gene of SARS-CoV-2 that adversely impacts detection by reverse transcription-PCR. J. Clin. Microbiol..

[16] Quick, J. & Barnes, K. nCoV-2019 sequencing protocol v3 (LoCost). https://www.protocols.io/view/ncov-2019-sequencing-protocol-v3-locost-bp2l6n26rgqe/v3 (2020).

[17] Tyson, J. R. *et al.* Improvements to the ARTIC multiplex PCR method for SARS-CoV-2 genome sequencing using nanopore. *bioRxiv Preprint Serv. Biol. *10.1101/2020.09.04.283077 (2020).

[18] Quick J (2016). Real-time, portable genome sequencing for Ebola surveillance. Nature.

[19] Simpson J. ncov-tools. http://github.com/jts/ncov-tools. Accessed 30 Sept 2021 (2021).

