# Author Correction: Emergence of a mutation in the nucleocapsid gene of SARS-CoV-2 interferes with PCR detection in Canada

**DOI:** 10.1038/s41598-022-17590-5

**Published:** 2022-08-19

**Authors:** Sandra Isabel, Mariana Abdulnoor, Karel Boissinot, Marc R. Isabel, Richard de Borja, Philip C. Zuzarte, Calvin P. Sjaarda, Kevin R. Barker, Prameet M. Sheth, Larissa M. Matukas, Jonathan B. Gubbay, Allison J. McGeer, Samira Mubareka, Jared T. Simpson, Ramzi Fattouh

**Affiliations:** 1 Department of Laboratory Medicine and Pathobiology, Temerty Faculty of Medicine, University of Toronto, Toronto, ON Canada; 2 Public Health Ontario, Toronto, ON Canada; 3 Division of Microbiology, Department of Laboratory Medicine, St. Michael’s Hospital, Unity Health Toronto, 30 Bond Street, Toronto, ON M5B 1W8 Canada; 4 Ontario Institute for Cancer Research, Toronto, ON Canada; 5 Queen’s Genomics Lab at Ongwanada (Q-GLO), Kingston, ON Canada; 6 Department of Psychiatry, Queen’s University, Kingston, ON Canada; 7 Division of Microbiology, Department of Laboratory Medicine and Genetics, Trillium Health Partners, Mississauga, ON Canada; 8 Department of Pathology and Molecular Medicine, Queen’s University, Kingston, ON Canada; 9 Division of Microbiology and Infectious Diseases, Kingston Health Sciences Center, Kingston, ON Canada; 10 Department of Biomedical and Molecular Sciences, Queen’s University, Kingston, ON Canada; 11 Hospital for Sick Children, Toronto, ON Canada; 12 Lunenfeld-Tanenbaum Research Institute, Sinai Health System, Toronto, ON Canada; 13 Sunnybrook Research Institute, Toronto, ON Canada; 14 Department of Computer Science, University of Toronto, Toronto, ON Canada; 15 Li Ka Shing Knowledge Institute, Unity Health Toronto, Toronto, ON Canada; 16 Institute for Better Health, Trillium Health Partners, ON Mississauga, Canada; 17 Matane, Quebec, Canada

Correction to: *Scientific Reports* 10.1038/s41598-022-13995-4, published online 27 June 2022

The original version of this Article contained an error in the spelling of the author Allison J. McGeer which was incorrectly given as Alisson J. McGeer.

In addition, the author Marc R. Isabel was incorrectly affiliated with “Department of Laboratory Medicine and Pathobiology, Temerty Faculty of Medicine, University of Toronto, Toronto, ON, Canada”. The correct affiliations are listed below.

Marc R. Isabel is an independent scientist.

Matane, Quebec, Canada

The original Article has been corrected.

